# Using causal models and theories to achieve equitable implementation science in global health

**DOI:** 10.1186/s44263-025-00203-4

**Published:** 2025-10-01

**Authors:** Olakunle Alonge

**Affiliations:** Sparkman Center for Global Health, University of Alabama at Birmingham, Suite 517C, Ryals Building, 1655 University Blvd, Birmingham, AL 35233 USA

Implementation science can advance efforts to decolonize global health. These efforts must however be guided by causal models and theories that integrate root causes, pathways, and mechanisms of health inequities as well as explicate established social justice and human rights theories as part of implementation research and practice.

## Background

Health inequalities are differences in health outcomes resulting from differences in biological, psychosocial, and behavioral risk factors of diseases, among different population or social groups [1]. Health inequities are unfair health inequalities [1]. The aspiration of implementation science (IS) – the study of methods to promote the adoption and integration of evidence-supported interventions (ESI) into routine health care and public health settings to improve population health – cannot be achieved without addressing health inequities [1].

While equity-focused implementation frameworks abound in literature, there is not a single established equity-focused implementation theory, and only a limited number of equity-focused implementation models exist [1, 2]. In this frame, theories refer to a set of explicitly stated universal explanations that can predict an outcome [3]. For example, the Social Cognitive Theory explains human behaviors as a product of personal and environmental factors and the behavior itself. Models seek to provide causal explanation for a phenomenon or steps on how to accomplish an outcome [3], including explicating theories, applied to specific population settings. For example, the Capacity Opportunity Motivation-Behavior (COM-B) Model explicates aspects of the Social Cognitive Theory and other behavioral theories to explain steps for influencing a specific behavior change and designing behavior change interventions. Frameworks depict relationships among related constructs that may influence a phenomenon – and do not necessarily provide causal explanations [3]. For example, the Theoretical Domains Framework provides a list of unique constructs to consider in designing behavior change interventions.

Most equity-focused implementation frameworks are useful for: 1) exploring the role of social determinants of health, e.g., the Framework for Equity-focused Implementation Research for Health Programs; 2) identifying contextual factors (facilitators and barriers) and strategies to support implementation in low resource settings and/or that affect socially disadvantaged groups, e.g., the Health Equity Implementation Framework; and 3) measuring implementation outcomes to assess the impact of ESI and strategies on health inequities, e.g., Equity Consideration of Reach, Adoption, Effectiveness, Implementation and Maintenance Framework [1, 2]. The few available equity-focused implementation models are useful for planning and adapting ESI to the needs of socially disadvantaged groups, e.g., the Transcreation Model, or comprise adaptation of behavioral models to improve individual-level health behaviors in specific settings, e.g., COM-B [2].

More equity-focused implementation causal models and theories are needed to unpack and operationalize the causal factors, pathways, and mechanisms of health inequities as well as explicate established social justice and human rights theories as part of IS. Such causal models will inform the development of multifaceted implementation strategies to address health inequities.

## A causal model of health inequities in implementation science

**Fig. 1:**
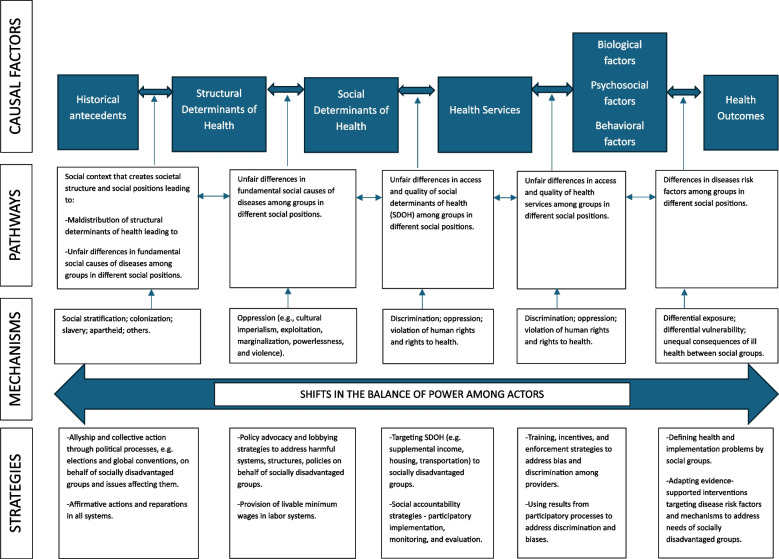
A causal model of health inequities in IS

In this article, a causal model of health inequities in implementation science (CAMHIS) is proposed drawing from health equity and implementation science literature, and the author’s research and graduate-level teaching experience in both fields. The model seeks to explicate the causal factors, pathways and mechanisms by which health inequities arise in any implementation context and identify evidence-supported implementation strategies for disrupting these pathways and mechanisms (Fig. 1).

### Causal factors of health inequities

Health inequalities, e.g., differences in maternal mortality rates due to pre-eclampsia comparing populations in different countries or racial groups within countries, are the starting point for defining health inequities. These inequalities must however be linked to specific underlying causal factors – differential access to and quality of health services and differences in social and structural determinants of health by population or social groups – to define them further as health inequities (Fig. 1). Social determinants of health are the conditions in which people are born, live and work; and structural determinants of health are the societal systems, structures, institutions, and policies that shape those conditions. Structural determinants of health are prescribed by the historical antecedents for a given population or setting. The notion of inequities derives primarily from the pathways and mechanisms by which these causal factors are linked, and whether these pathways and mechanisms are unfair or not. For example, differences in maternal mortality rates among racial groups linked to differences in living conditions and access to and quality of maternal health services which are in turn linked to racialized sociopolitical systems and policies that unfairly treat a racial group compared to another are health inequities. This is especially true if there are historical antecedents, e.g., history of colonization and racial supremacy, that prescribe those unfair systems and policies. Hence, IS focused on addressing health inequities needs to consider specific causal factors beyond the proximal risk factors of diseases, including differential access to and quality of health services and differences in social and structural determinants of health by social groups linked to their historical antecedents in a defined setting, and theorize on the pathways linking these factors.

### Pathways of health inequities

Pathways of health inequities show how differential experiences are linked within and between different causal factors to produce observed differences in health outcomes [4]. Drawing from social justice and human rights theories [5], pathways showing linked differences in access to and quality of health services, social and structural determinants of health among social groups are unfair if the distribution of these factors is not consistent with the view of justice by most members of a well-defined society. For example, the Rawl’s theory of social justice suggests that most members of a given society, blinded to the knowledge of their status in any social hierarchy, might regard the distribution of socioeconomic advantages as unfair if it further disadvantages those worse-off within that society [5]. Implicit within these theories is the fundamental idea that unfairness originates from a social context that creates societal hierarchies that stratify populations into different social positions and groups to systematically disadvantage certain groups [6]. This type of social context may facilitate maldistribution of structural determinants of health along with certain resources that individuals can use to minimize the impact of disease risk factors, including knowledge, financial, social, and physical capital (aka the fundamental social causes of diseases). It is important to recognize that such social contexts are actively constructed by political actors in any society – and partially imposed by external actors on societies in the Global South. Hence, pathways of health inequities in understanding efforts to decolonize global health through IS must originate from the historical antecedents while considering current political construction that shapes the social contexts in such societies (Fig. 1).

The pathways in Fig. 1 are depicted with bidirectional arrows to indicate that simultaneous causation exists between these factors. Hence, multiple causal pathways of health inequities are plausible for a defined health inequality within a specific implementation context.

### Mechanisms of health inequities

Mechanisms of health inequities are explicit and implicit processes by which differential experiences with a specific causal factor may occur. Specific mechanisms are plausible for explaining causal pathways of health equities. For example, social stratification [6], the process of arranging population groups in society into hierarchy based on their physical and psychological attributes and/or perceived status, and colonization have shaped political systems in many countries in the Global South as well as the power relations that govern the distribution of knowledge and resources for global health within and between countries. Unpacking how these mechanisms lead to maldistribution of structural determinants of health among social groups might yield higher-order and multifaceted implementation strategies to address health inequities across multiple implementation contexts. Similarly, discriminatory and oppressive practice*s *that systematically disadvantage a social group relative to another while delivering health services (e.g., within provider-patient encounters) may account for differential vulnerability to proximal risk factors of diseases and differences in health outcomes among social groups [7]. Hence, implementation strategies addressing these discriminatory and oppressive practices could be packaged across multiple programs and health services delivery activities, invariant of the specific ESI, to address health inequities in any implementation context.

### Implementation strategies for reducing health inequities

At the heart of all plausible mechanisms for explaining health inequities are two important theses. First, these mechanisms occur at the interface of encounters between actors (e.g., colonizer vs. colonized, socially advantaged vs. socially disadvantaged, implementers vs. beneficiaries) [1]. Second, power, “the ability to shape the thinking and/or actions of other actors” [7] is asymmetrical and fluid between these actors and shapes the distribution of causal factors of health inequities [1]. Hence, implementation strategies must address these power asymmetries to address health inequities. There are a number of evidence-supported implementation strategies that can address power asymmetries along the pathways and mechanisms of health inequities explicated thus far [1]. For example, reparations and collective action on behalf of socially disadvantaged groups might be conceptualized as implementation strategies to address vestiges of colonialism – leading to significant changes in the structural and social determinants of health to reduce health inequities. The Colombian Victim Law program is an example of such reparation programs [8]. It provided cash reparations, housing subsidies, and land restitution along with other multi-sector support to victims of armed conflicts committed by state forces; and has been linked to improved health due to better social determinants of health [8]. Similarly, social accountability strategies, including participatory monitoring by members of socially disadvantaged groups, e.g., through community scorecards, might be deployed to address unfair differences in access to and quality of health services because of discrimination and violation of rights to health to reduce health inequities. For example, social accountability initiatives were credited with influencing the structural determinants of health, improving quality of health services, trust, and power relations to address inequities of maternal health outcomes in Gujarat, India [9]. It is important to distinguish and conceptualize these inequities-reducing strategies in addition to the more traditional implementation strategies (e.g., training and supervision strategies) usually specified to effectively deliver an ESI to more intentionally address health inequities through IS.

## Data Availability

No datasets were generated or analysed during the current study.
